# Monocyte and macrophage foam cells in diabetes-accelerated atherosclerosis

**DOI:** 10.3389/fcvm.2023.1213177

**Published:** 2023-06-12

**Authors:** Jocelyn Cervantes, Jenny E. Kanter

**Affiliations:** Department of Medicine, UW Medicine Diabetes Institute, University of Washington, Seattle, WA, United States

**Keywords:** monocytes, diabetes, macropahge, foam cell, triglyceride-rich lipoprateins

## Abstract

Diabetes results in an increased risk of atherosclerotic cardiovascular disease. This minireview will discuss whether monocyte and macrophage lipid loading contribute to this increased risk, as monocytes and macrophages are critically involved in the progression of atherosclerosis. Both uptake and efflux pathways have been described as being altered by diabetes or conditions associated with diabetes, which may contribute to the increased accumulation of lipids seen in macrophages in diabetes. More recently, monocytes have also been described as lipid-laden in response to elevated lipids, including triglyceride-rich lipoproteins, the class of lipids often elevated in the setting of diabetes.

## Introduction

Foam cells are a hallmark of atherosclerosis which is the underlying pathology in atherosclerotic cardiovascular disease (ACVD) that results in myocardial infarction and stroke. These lipid-laden cells are named after their foamy appearance due to the increased uptake of pro-atherogenic lipids. Many lesional foam cells are derived from macrophages, but other cell types, such as vascular smooth muscle cells, also readily become foam cells (1), a topic discussed in other excellent reviews (2, 3). It has long been recognized that modified, but not native, low-density lipoprotein (LDL) is excessively taken up by macrophages (4), and LDL is in the causal pathway of atherosclerosis. Still, other lipids such as very low-density lipoprotein (VLDL) and their remnants and lipolysis products can also induce lipid loading (5), which may become all the more important in conditions where those classes of lipids are elevated. One such condition is diabetes. Diabetes, both type 1 and type 2, results in about a 2-fold increase in atherosclerotic cardiovascular disease (6, 7), and in this mini-review, we will discuss if part of the increased risk could be driven by augmented monocyte and macrophage foam cell formation.

## Foam cells in diabetes-accelerated atherosclerosis

### Macrophage foam cell in diabetes

**Figure 1:**
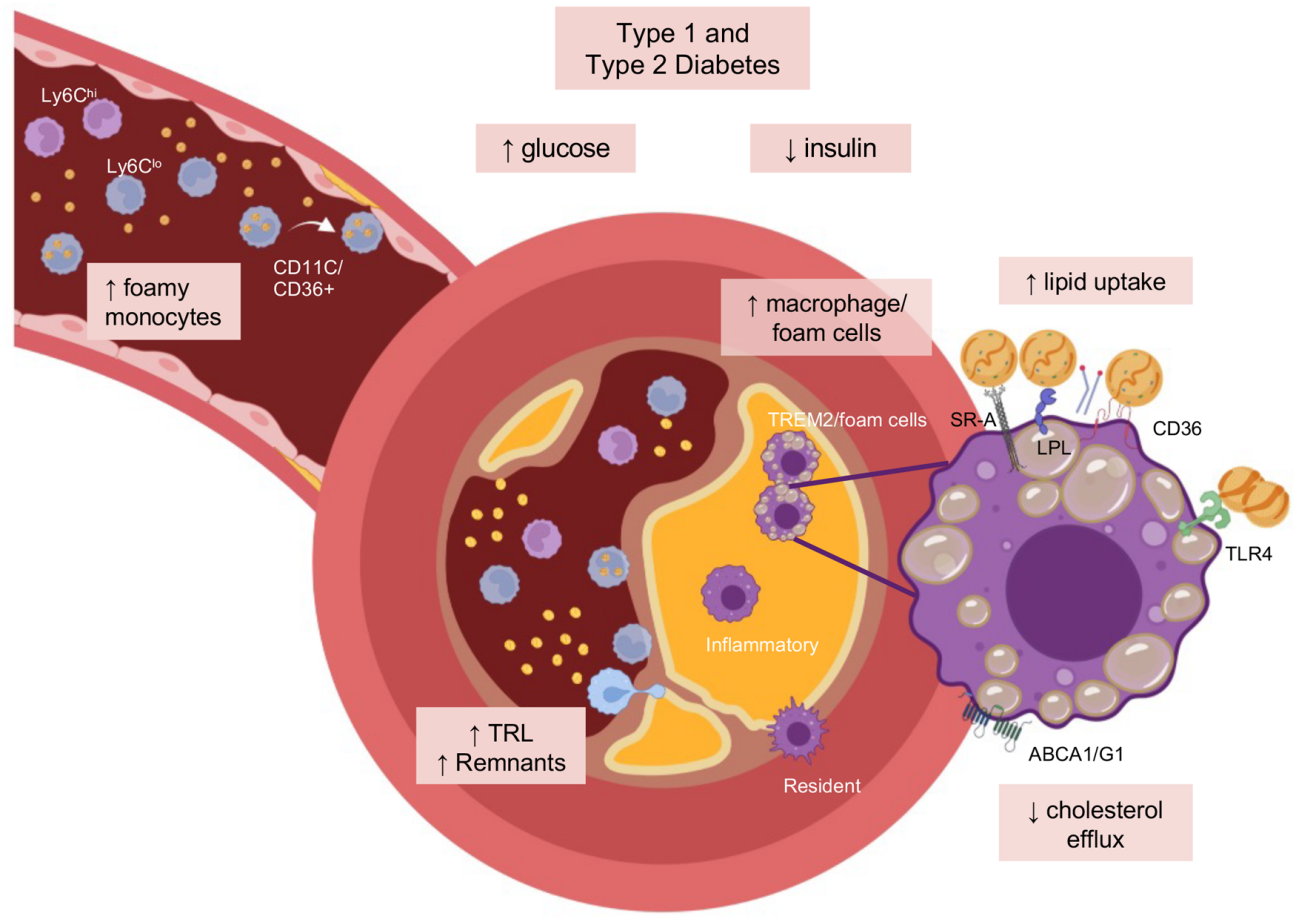
Monocyte and macrophage foam cells in diabetes. Foam cells may partly contribute to diabetes-accelerated atherosclerosis through changes in the myeloid cell compartment. In the setting of diabetes, both hyperglycemia and insulin insufficiency has been linked with increased monocyte lipid loading, macrophage lipid uptake, and cholesterol efflux. The effects of diabetes may, in part, be driven by increases in the expression of receptors that mediate lipid uptake, such as CD36, SR-A, LOX-1, and LPL (lipoprotein lipase), and or reductions in cholesterol efflux transporter (ABCA1, ABCG1). TLR4 can mediate the uptake of aggregated LDL. In addition, diabetes increases circulating and artery wall levels of triglyceride-rich lipoproteins (TRL) and their remnants, which are highly atherogenic. Figure created in part using BioRender.

Intracellular lipid accumulation, or foam cell formation, results from increased uptake relative to metabolism and efflux of said lipids. Like other cells, macrophages store excess lipids as lipid droplets within the cytoplasm, giving the cells their characteristic foamy appearance. As stated above, we know that diabetes results in increased atherosclerotic cardiovascular disease (6), and post-mortem analysis of atherosclerotic lesions indicate that diabetes (both type 1 and type 2) is associated with increased macrophage accumulation and necrotic core burden (8, 9), making it tempting to hypothesize that macrophages and macrophage foam cells are key players in the acceleration of atherosclerosis seen in diabetes (Figure 1). We have shown that in a model of type 1 diabetes, which results in accelerated atherosclerosis (10), diabetes results in increased macrophage cholesteryl ester accumulation (11, 12). Similar findings have been reported in human monocyte-derived macrophages from people with type 2 diabetes (13), suggesting that part of the accelerated atherosclerotic phenotype could be due to increased macrophage lipid accumulation.

Diabetes is a metabolic disease that results from a lack of (type 1 diabetes) or insufficient insulin production and insulin signaling (type 2 diabetes), resulting in increased blood glucose. Insulin also significantly affects lipid metabolism, and people with less well-controlled type 1 diabetes or type 2 diabetes often have elevated levels of triglyceride-rich lipoproteins (VLDL and chylomicrons) and their partially metabolized remnants, which are believed to be highly atherogenic (14). The question remains, is the increased cholesteryl ester accumulation in macrophages in diabetes due to increased plasma lipids levels, or is there an effect of diabetes on macrophages allowing for augmented lipid loading, which may contribute to accelerated atherosclerosis? Primary macrophages isolated from models of type 1 and type 2 diabetes demonstrate that diabetes augments the uptake of exogenously added oxidized LDL (15, 16), arguing that diabetes may alter macrophages resulting in increased lipid uptake and or reduced efflux.

### Diabetes augments lipid uptake

Macrophage lipid uptake of modified LDL is mediated by scavenger receptor (SR)-mediated endocytosis, phagocytosis, and micropinocytosis (17). The most studied scavenger receptors are cluster of differentiation 36 (CD36), SR-A, and lectin-like oxLDL receptor-1 (LOX-1), all of which have been shown to contribute to foam cell formation and atherosclerosis, at least in part (18–24). In addition to the traditional SR-mediated uptake pathways, toll-like receptor 4 (TLR4) has been suggested to be involved in the uptake of aggregated LDL (25, 26), which may also contribute to foam cell formation. Early studies suggested that CD36 expression was more common in endarterectomy specimens from people with reported hyperglycemia (27). This observation was followed by investigating whether elevated glucose induces CD36 expression. Indeed, human monocyte-derived macrophages exposed to elevated glucose in the media displayed increased cell surface expression of CD36 via effects on translation rather than on transcription (27). Similar findings have been replicated in other myeloid cell systems, demonstrating that increased glucose concentrations can increase oxidized LDL uptake (16). Forced uptake of glucose via overexpression of the glucose transporter (GLUT1) results in increased CD36 expression and increased cholesterol accumulation in macrophages overexpressing GLUT1 compared to control cells expressing an empty vector (28), potentially suggesting that the hyperglycemia observed in diabetes primes the macrophages to take up more lipids by increasing their expression of CD36.

Similarly, defective insulin signaling within macrophages results in increased CD36 expression, again via a mechanism largely independent of increased transcription (29). Furthermore, both SR-A and LOX-1 mRNA and protein are increased in macrophages from mice with diabetes, again via mechanisms that point toward glucose being the culprit (30, 31), thus suggesting that the increase in lipid loading observed in diabetes may be in part due to intrinsic effects on uptake pathways which may be driven by hyperglycemia and or defective insulin signaling.

Alternatively, or perhaps in conjunction with the receptors mentioned above, triglyceride-rich lipoproteins may be acted upon locally in the artery wall by lipoprotein lipase (LPL), resulting in free fatty acids and remnant lipoproteins, both of which may result in lipid uptake and lipid loading. Diabetes has been shown, at least in some studies, to increase macrophage LPL (32). LPL deficiency selectively in macrophages results in reduced lipid loading in response to VLDL and concomitant reduced atherosclerosis (33).

### Impairment of macrophage cholesterol esterification and cholesterol efflux in diabetes

Once lipoproteins are taken up into the cell, they are hydrolyzed by lysosomal acid lipase (LIPA). Interestingly, genome-wide association studies have indicated that the atherosclerotic risk allele for LIPA is associated with increased enzyme activity (34), suggesting a pro-atherosclerotic role for intracellular lipolysis of lipids. Cholesterol then undergoes re-esterification, which in macrophages primarily is accomplished by Acyl-CoA: cholesterol acyltransferase 1 (ACAT1). ACAT1-deletion, selectively in the hematopoietic compartment or macrophages, reduces cholesteryl ester accumulation and reduced atherosclerosis in most studies (35, 36), but not all (37). There are some suggestions that ACAT1 might be increased in the setting of diabetes. Teasaki et al. demonstrate that macrophages from mice with diabetes and monocyte-derived macrophages from people with type 1 diabetes had increased expression of *ACAT1* (38), potentially suggesting that cholesterol esterification may be increased in diabetes too.

Conversely, cholesterol removal from cells largely depends on cholesterol efflux mediated via the ATP-binding cassette transporters A1 and G1 (ABCA1 and ABCG1) to apolipoprotein A1 (APOA1) and high-density lipoproteins (39). We and others have demonstrated that levels of both ABCA1 and ABCG1 are reduced, and efflux to APOA1 is impaired in macrophages isolated from mouse models of type 1 and type 2 diabetes (12, 13, 15), suggesting that reduced efflux of cholesterol might contribute to the increased lipid loading observed in macrophage in diabetes.

### Consequences of lesional foam cell formation in diabetes

A key question remains; are foam cells really in the causal pathway of atherosclerosis, and in that case, how? Most studies show a clear correlation between macrophage lipid loading and atherosclerosis, indicating that this is the case. However, pathways that alter macrophage lipid uptake and handling often result in other changes in monocyte and macrophage behavior. For example, reduced cholesterol efflux or inability to esterify intracellular cholesterol increases monocyte levels in circulation. Deletion of either ABCA1 and/or ABCG1 dramatically enhances hematopoietic stem cell proliferation and results in monocytosis (40). Similarly, ACAT1-deficiency in the hematopoietic compartment changes circulating monocyte levels (35). Since monocytosis is thought to contribute to the accelerated atherogenesis (41, 42), it becomes difficult to assess the relative contribution of efflux or cholesterol esterification vs. monocytosis in atherogenesis.

Furthermore, receptors such as CD36 that contribute to lipid loading have other effects independent of their roles in lipid uptake, such as mediating sterile inflammation and migration, for example (43, 44). Additionally, oxidized phospholipids on LDL that mediate the lipid uptake and accumulation are thought to contribute to macrophage inflammation (45, 46). Some of the pro-inflammatory effects have been attributed to CD36 acting as a co-receptor alongside toll-like receptors 2 and 6 (43, 47), perhaps making it difficult to unequivocally conclude that foam cells *per se* play a role in atherosclerosis, based on data with deficiencies in those receptors. The idea that lipids and lipid loading induces inflammatory changes in macrophages and that this, in part, is what drives atherogenesis has recently been put into question. Work from the Glass laboratory (48) and recent data from atherosclerotic lesion macrophages suggest that lipid loading does not necessarily induce a proinflammatory state (49). At least three different macrophage populations have been identified in the aortas using single-cell RNA sequencing (scRNA-seq); the resident, the inflammatory, and TREM2^hi^ (triggering receptor expressed on myeloid cells 2) macrophages (49–52). Interestingly, it is not the inflammatory macrophages but rather the TREM2^hi^ macrophages that are lipid-laden or foamy (49). These inflammatory and foamy macrophage populations were first identified in mouse lesions, but similar inflammatory and lipid-associated clusters have since been detected in human atherosclerotic lesions (53–55). To date, no global transcriptomic analyses have been carried out on atherosclerotic lesions comparing non-diabetic and diabetic lesions, but these will surely aid in our understanding of how diabetes accelerates atherosclerosis and what effects diabetes has on myeloid cells.

Another approach to interrogate whether lipid loading plays a causal role in atherosclerosis was employed by Paul et al. (56). Lipid loading induces the expression of lipid droplet-associated proteins such as perilipin 2, and perilipin 2 is known to promote lipid droplet formation. Notably, deleting said lipid droplet protein restricts lipid loading in macrophages and subsequently reduces atherosclerosis (56), suggesting that foam cells are in the causal pathway.

Lesional macrophages also contribute to the expansion of the necrotic core, a key feature often associated with unstable atherosclerotic lesions. Excess uptake of lipoproteins can result in macrophage cell death, which is believed to contribute to necrotic core expansion. Both human studies (8) and our work using mouse models (11, 57) suggest that diabetes results in an augmented necrotic core expansion. One of the pathways proposed to induce macrophage cell death in response to lipids is pyroptosis. However, very recently, we could demonstrate that hematopoietic deficiency of a key protein involved in pyroptosis, Gasdermin D, did not alter necrotic core expansions in response to diabetes, arguing that other pathways are involved in driving necrotic core expansion in diabetes (58). One such pathway may be driven by the dyslipidemia often observed in diabetes that increases triglyceride-rich lipoproteins and their remnants. These remnants accumulate in the artery wall and are in close proximity to lesional macrophages (11, 57, 59). Suppression of plasma triglyceride-rich lipoproteins and their remnants blocks the necrotic core expansion associated with diabetes, suggesting a link between dyslipidemia seen in diabetes and the increase in the unstable lesional phenotype (11, 57). Furthermore, if monocyte recruitment is impaired, the expansion of the necrotic core is blunted under diabetic conditions (60) arguing that monocytes and macrophages contribute to the enlarged necrotic core in response to diabetes.

### Monocyte foam cells

Where do these lesional foam cells come from? Although local proliferation of resident intimal macrophages occurs early in atherosclerosis development, where they contribute to foam cell formation and early atherosclerosis (61, 62), the progression of atherosclerosis appears to be driven by the recruitment of monocyte-derived cells (61). Circulating monocytes are derived from bone marrow progenitor cells, and similar to humans, mice have two major subtypes of monocytes in circulation. Murine monocytes are distinguished based on the surface expression of the lymphocyte antigen 6 complex locus C (Ly6C), whereas human monocytes are separated based on their surface expression of CD14 and CD16. Ly6C^hi^ are classical monocytes (CD14^hi^CD16^–^ in humans) that are critical in acute inflammatory responses, while Ly6C^lo^ monocytes (CD14^–^CD16^+^ in humans) have been described as non-classical, patrolling monocytes that play an important role in vascular homeostasis. Both Ly6C^lo^ and Ly6C^hi^ monocytes can employ different receptors to exit the circulation and enter the artery wall, where they mature into macrophages within lesions (2, 61, 63). More recently, it was demonstrated that Ly6C^lo^ monocytes might play an atheroprotective role via an increased patrolling behavior, which was mediated by CD36 and could be induced by modified LDL (64).

Studies have indicated that monocytes, similar to macrophages, become lipid loaded in response to hypercholesterolemia and hypertriglyceridemia (65, 66). Data from our lab suggests that monocytes become lipid loaded in response to diabetes (57). Lipid-laden circulating monocytes have also been reported in individuals with metabolic syndrome (67). The increase in foamy monocytes correlates with fasting plasma triglyceride levels in these individuals, and following a high-fat meal, foamy monocytes are further increased in circulation, suggesting that circulating monocytes acutely respond to changes in triglyceride-rich lipoproteins.

The monocyte population that becomes lipid-laden primarily expresses CD11C and CD36 and upon exposure to cholesteryl ester-rich VLDLs, monocytes can mature towards CD11C^+^ monocytes in circulation (67, 68). These CD11C^+^/CD36^+^ are most likely the same non-classical patrolling monocytes, as CD36 is primarily expressed in this population in both mice and humans (69). Tracing studies confirm that these CD11C^+^ foamy monocytes infiltrate nascent lesions, and CD11C ablation prevents their accumulation in early atherosclerotic lesions (65). Neutral lipid accumulation in monocytes can alter their migratory capacity (66). One could speculate that excessive lipid loading of Ly6C^lo^ monocytes changes their patrolling activity, so they are no longer atheroprotective.

What lipids are monocytes responding to? In experiments using poloxamer 407, Saja et al. showed that although the lipase inhibitor resulted in dramatic elevations of triglyceride-rich lipoproteins, which increased the crawling of non-classical monocytes in circulation and their extravasation into the heart, it did not result in significant monocyte lipid loading, arguing that monocytes are unable to take up these very large triglyceride-rich particles (70), potentially arguing that it is the smaller remnants that are taken up, or lipolysis products (71). Consistent with that idea of remnants inducing monocyte lipid loading, we recently demonstrated that in a model of type 1 diabetes, there is a dramatic increase Ly6C^lo^ monocyte side-scatter (a marker of lipid accumulation (66)) which was reduced when remnant lipoprotein levels were reduced (57). This was associated with reduced diabetes-accelerated atherosclerosis. At this point, whether these monocyte foam cells contribute to lesional foam cell formation and to diabetes-accelerated atherosclerosis is unexplored.

### Does suppressing foam cells by reducing triglyceride-rich lipoproteins prevent diabetes-accelerated atherosclerosis?

Current approaches to lower plasma lipids in people with and without diabetes mainly include statins and proprotein convertase subtilisin/kexin type 9 (PCSK9) inhibitors, primarily affecting LDL cholesterol. Despite lowering LDL cholesterol, diabetes still results in about a 2-fold increased risk of cardiovascular disease (6), suggesting additional pathways are at play. Remnant lipoproteins have been hypothesized to mediate this residual risk in people with diabetes (7). Remnants are particularly atherogenic, partly because they carry more cholesterol per particle than LDL and, even in their native form, induce cholesterol accumulation in macrophages (72) and potentially monocytes (57). As of yet, there are no approved therapies that successfully target triglyceride-rich lipoproteins and remnants, which are often elevated in diabetes, but inhibition of Apolipoprotein C3 (APOC3) or Angiopoietin-like 3 (ANGPTL3) has, in clinical trials, been shown to reduce triglycerides and triglyceride-rich lipoproteins (73–76). For example, antisense oligonucleotide-mediated suppression of APOC3 resulted in an almost 70% reduction in plasma triglycerides in people with type 2 diabetes (77).

We have recently demonstrated APOC3 silencing using an antisense approach results in a dramatic reduction in triglycerides and diminished diabetes-accelerated atherosclerosis (11). This was associated with a dramatic reduction in diabetes-augmented macrophage cholesteryl ester accumulation, suggesting that lowering remnants reduces macrophage foam cells. Silencing ANGPTL3 also reduced atherosclerosis in several models of atherosclerosis in non-diabetic mice (74, 76), but nothing is known about whether reducing ANGPTL3 would rescue diabetes-accelerated atherosclerosis or foam cell formation.

## Discussion

As discussed above, macrophage foam cells are a key characteristic of atherosclerosis, and diabetes appears to augment this feature. Diabetes creates a perfect storm by inducing changes to monocytes and macrophages, priming them for increased lipid uptake and reduced ability to efflux cholesterol while also resulting in elevated circulating lipid levels, thus resulting in increased monocyte and macrophage lipid loading (Figure 1). More research is needed to fully understand the role of monocyte and macrophage foam cells in diabetes-accelerated atherosclerosis and how best to reverse it. In addition to the studies mentioned above lowering plasma triglyceride-rich lipoproteins as an approach to reduced diabetes-augmented atherosclerosis, many studies have also pharmacologically targeted uptake or efflux of lipids (78). Intriguingly, foam cells have also been reported in other complications of diabetes, such as diabetic kidney disease (79). Therefore, it is tempting to speculate that augmented foam cell formation is a common theme in diabetes and that reducing foam cell formation in diabetes may also benefit other organs.
